# Effect of Variations in Gap Junctional Coupling on the Frequency of Oscillatory Action Potentials in a Smooth Muscle Syncytium

**DOI:** 10.3389/fphys.2021.655225

**Published:** 2021-10-01

**Authors:** Shailesh Appukuttan, Keith L. Brain, Rohit Manchanda

**Affiliations:** ^1^Department of Biosciences and Bioengineering, Indian Institute of Technology Bombay, Mumbai, India; ^2^Institute of Clinical Sciences, College of Medical and Dental Sciences, University of Birmingham, Birmingham, United Kingdom

**Keywords:** syncytium, action potential, gap junction, oscillation, frequency

## Abstract

Gap junctions provide pathways for intercellular communication between adjacent cells, allowing exchange of ions and small molecules. Based on the constituent protein subunits, gap junctions are classified into different subtypes varying in their properties such as unitary conductances, sensitivity to transjunctional voltage, and gating kinetics. Gap junctions couple cells electrically, and therefore the electrical activity originating in one cell can affect and modulate the electrical activity in adjacent cells. Action potentials can propagate through networks of such electrically coupled cells, and this spread is influenced by the nature of gap junctional coupling. Our study aims to computationally explore the effect of differences in gap junctional properties on oscillating action potentials in electrically coupled tissues. Further, we also explore variations in the biophysical environment by altering the size of the syncytium, the location of the pacemaking cell, as well as the occurrence of multiple pacemaking cells within the same syncytium. Our simulation results suggest that the frequency of oscillations is governed by the extent of coupling between cells and the gating kinetics of different gap junction subtypes. The location of pacemaking cells is found to alter the syncytial behavior, and when multiple oscillators are present, there exists an interplay between the oscillator frequency and their relative location within the syncytium. Such variations in the frequency of oscillations can have important implications for the physiological functioning of syncytial tissues.

## Introduction

Cells in certain kinds of tissues, such as cardiac and smooth muscle, are known to be electrically coupled to adjacent cells, thereby forming an electrical syncytium (Eisenberg et al., 1979). These intercellular connections are formed by means of protein structures known as gap junctions. Gap junctions provide pathways between cells which allow exchange of ions and small metabolites between the connected cells (Meşe et al., 2007). By virtue of this intercellular coupling, electrical activity in a syncytium, such as action potentials (APs), that originate in any cell can potentially be propagated to other cells in its vicinity. The extent of spatial spread is determined by various factors such as the unitary conductance, sensitivity to transjunctional voltage and gating kinetics. These factors are known to vary based on the constituent protein sub-units, known as connexins, forming the gap junction. Connexins are known to exist in several forms, and thus there exists a wide variety of gap junction subtypes which vary in their biophysical properties based on the configuration of the constituent connexins (Koval et al., 2014). They are broadly classified as homomeric-homotypic, heteromeric-homotypic, homomeric-heterotypic or heteromeric-heterotypic (Vogel and Weingart, 2002), based on the nature of the hemi-channels forming the gap junction. Homomeric refers to the presence of only a single connexin subtype in a hemi-channel, as opposed to heteromeric indicating multiple connexin subtypes, and homotypic refers to both hemi-channels being identical, in contrast to heterotypic where the two hemi-channels are different. Within each of these classes, there exists a wide diversity in biophysical behavior based on the nature of the specific connexins involved. These differences in the nature of intercellular coupling between cells holds huge potential to influence tissue functioning.

Certain cells are known to exhibit pace-making activity, whereby they produce APs at regular time intervals. This periodic activity drives electrical activity in other cells within the syncytium. An example of this is the sinoatrial (SA) node in cardiac tissue, which is known to exhibit pace-making activity by producing APs at regular intervals (DiFrancesco, 1993). Pacemaking activity has also been reported in various smooth muscle tissues, such as the gastrointestinal tract (Sanders et al., 2006) and the urinary bladder wall (Coolsaet, 1985). The origin of pacemaking activity and their physiological relevance in certain smooth muscle tissues is still inconclusive. For example, the detrusor does not possess well-defined pacemaking centers such as in the cardiac system. Also, unlike the cardiac tissue where the pacemaking activity results in the synchronous excitation and contraction of the entire organ, in smooth muscle tissues these may also underly more localized contractions, such as the segmentation motor pattern observed in the intestine (Huizinga and Chen, 2014).

All of the above tissues are known to form electrical syncytia, wherein the electrical response of an individual cell is influenced by a host of syncytial features. These include the characteristics of coupling between cells, the biophysical properties of neighboring cells, and the arrangement of cells within the syncytium. The nature of intercellular coupling is determined by the gap junction subtype connecting the two cells. These subtypes are physiologically unique, and any subtype often cannot be adequately replaced by another subtype (Desplantez et al., 2007). In the current study, we aim to investigate various interactions, such as: (i) the effect of the extent of gap junctional coupling on pacemaking or oscillatory activity of cells, (ii) the effect of variations in the gap junction subtypes on the frequency of the produced oscillations, (iii) the effect of syncytial size on exhibited oscillation frequency, and (iv) the influence of the location of the oscillator on syncytial behavior. As a representative subset of the wide diversity in gap junctions, we have focused on three homomeric-homotypic subtypes — Cx40, Cx43, and Cx45. Experimental studies have reported their presence in syncytial tissues such as cardiac and detrusor smooth muscle (DSM) layer of the urinary bladder wall (Darrow et al., 1995; Haefliger et al., 2002). They are also known to possess widely differing biophysical properties, and thus prove ideal for our investigation.

## Materials and Methods

**FIGURE 1:**
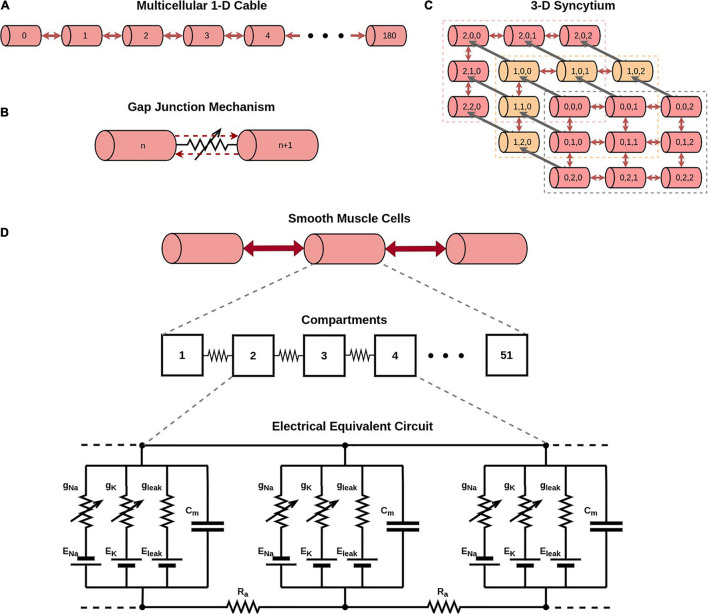
Overview of model development. **(A)** Illustration of a 1-dimensional chain of 181 cells forming a long multi-cellular cable. **(B)** Simplified representation of the gap junction mechanism employed in NEURON simulator. **(C)** Illustration of a 3-dimensional syncytium consisting of 27 cells organized as a cube of size 3. In our study we have explored syncytia of various sizes. Do note that different colors have been employed for purposes of clarity in illustration, and do not signify differences in biophysical features. **(D)** Illustration of the compartmental modeling technique as applied to the model employed in our study. Each smooth muscle cell in our model is modeled to consist of 51 segments. The electrical equivalent circuit for the cell membrane of three such adjacent compartments, along with their interconnection, is depicted in the bottom panel. Each compartment has Hodgkin-Huxley channels, thereby enabling them to produce action potentials.

Simulations were conducted on the NEURON simulation environment, a compartmental modeling platform (Hines and Carnevale, 2001). An overview of the compartmental modeling technique and equations underlying it have been presented in Supplementary Material 1. We have employed models with two distinct morphologies in the present study: (i) a 1-D model of DSM cells, consisting of a long chain of 181 cells; the total number of cells was chosen such as to eliminate reflection at the ends when stimulus is applied at the central cell, and (ii) a 3-D syncytial model consisting of DSM cells arranged in a cubic lattice layout; different sizes of the syncytia were explored in this study. This is illustrated in Figure 1, with each individual cell being multi-compartmental so as to provide spatial resolution. Recordings of membrane potential were always obtained from the central compartment of the specified cell. The development of these models is detailed in Appukuttan et al. (2015a), and described briefly in Supplementary Material 2.

The original models are purely passive, with no active ion channels. The cells were enhanced with the incorporation of AP generation mechanisms by endowing them with the classical Hodgkin-Huxley (HH) channels. These comprize of sodium (Na), potassium (K) and leakage (L) channels, and their conductances and currents are defined as follows:


(1) $$\begin{array}{ccc} g_{Na} & = & {\overset{¯}{g}}_{Na}\times m^{3}\times h\\ g_{K} & = & {\overset{¯}{g}}_{K}\times n^{4}\\ I_{X} & = & g_{X}\times \left(V_{m}-E_{X}\right) \end{array}$$

where ${\bar{g}}_{N\,a}$ and ${\bar{g}}_{K}$ are the peak sodium and potassium conductances, respectively, *g*_*Na*_ and *g*_*K*_ are the instantaneous sodium and potassium conductances, respectively, *m* and *h* are the activation and inactivation parameters, respectively, for sodium channels, *n* is the activation parameter for potassium channels, *g*_*L*_ is the constant leakage conductance, *V*_*m*_ is the membrane potential, *I*_*X*_ is the current flowing through channel X (X referring to Na, K, and leak channels), and *E*_*X*_ is its reversal potential.

Detrusor smooth muscle is known to exhibit action potentials of diverse temporal profiles (Appukuttan et al., 2018). The short duration APs are termed as spike-type, while the long duration APs are termed as pacemaker-type (Meng et al., 2008; Hayase et al., 2009). These are not observed as two distinct categories, but the DSM rather exhibits a wide variety of gradients in the elicited AP shapes. It is thus desirable to employ and explore APs of different temporal profiles.

The Hodgkin-Huxley (HH) channels produce an AP of ∼5 ms duration. Additionally, to obtain APs of wider time courses, the classical HH mechanism was modified by varying the Q_10_ temperature coefficient parameter (see Table 1), as demonstrated previously (Appukuttan and Manchanda, 2016). These wider AP generating mechanisms have been termed as HH-50 (AP with ∼50 ms duration) and HH-500 (AP with ∼500 ms duration). It is important to highlight that the model does not contain any other temperature dependent component. Therefore, the only effect of changing the temperature in these simulations is solely restricted to the Hodgkin-Huxley channels employed in the model. More specifically, the temperature coefficient parameter alters the time constants associated with parameters *m*, *n*, and *h*.

**TABLE 1:** Action potentials of different time courses obtained by adjusting Q_10_ temperature coefficient parameter in the classical HH model.

AP type	AP duration (ms)	Q_10_
HH	5.1	1
HH-50	50.4	0.0556
HH-500	511.2	0.00524

*Source: Appukuttan and Manchanda (2016).*

**FIGURE 2:**
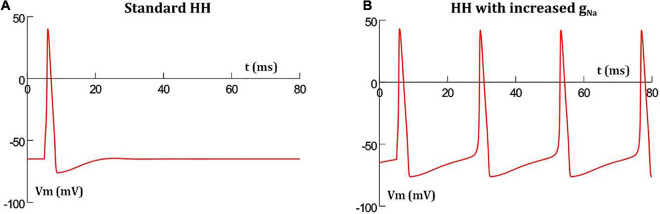
Hodgkin-Huxley action potential profile in a single isolated cell **(A)** with standard parameter values produces a single AP when stimulated, but thereafter settles back to resting potential, **(B)** with increased maximum sodium conductance, the short initial stimulus triggers the first AP, and thereafter enters into oscillations without any further external stimuli.

It has been reported that increasing the conductance of sodium channels in the HH mechanism results in oscillatory behavior (Horng and Huang, 2006). This is demonstrated in Figure 2 where the classical HH AP (Figure 2A) produces oscillations when the maximum sodium conductance was increased from 0.12 to 0.19 S/cm^2^ (Figure 2B). This was the minimal increase required in a model of a single isolated detrusor cell to produce oscillatory behavior. In our 1-D and 3-D models, a larger increase in the maximum sodium conductance was required to produce oscillations owing to the presence of gap junctions acting as shunts. This increase was incorporated only in the centrally located cell in the 1-D chain of cells, thereby endowing it with pacemaking ability, and in the oscillator cells, as described later, for the 3-D syncytial model. For each simulation, the AP frequency was measured by averaging the interval between the 3rd and 6th spike, thereby taking into account the time initially required for the model parameters to stabilize; a common protocol for simulations run on NEURON simulator.

Gap junctional coupling mechanisms for Cx40, Cx43, and Cx45 homomeric-homotypic gap junctions were developed. These are known to differ in their sensitivity to transjunctional voltage, unitary conductances and gating kinetics (Desplantez et al., 2004, 2007). Their modeling, incorporating the above differences, has been presented in an earlier study (Appukuttan et al., 2015b) and primarily involved constructing mathematical functions for hemi-channels to provide best fit to experimentally reported data. This process involved utilizing experimentally reported values of the main and residual state conductance levels of each gap junction subtype (g_*main*_ and g_*residual*_, respectively), their sensitivity to transjunctional voltage, and the kinetics of gap junctional inactivation and recovery from inactivation (Moreno et al., 1995; Lin et al., 2003; Desplantez et al., 2004, 2007). The development of these gap junctional models is elaborated in Supplementary Material 3.

## Results

We first present the effect of variations in the extent of gap junctional coupling between cells on the frequency of oscillations, irrespective of the gap junction subtype. This is followed by an evaluation of the influence of various gap junction subtypes on oscillatory behavior. Further, we present the influence of the size of the syncytium, and of the relative locations of the oscillators on syncytial behavior.

### Variations in the Extent of Coupling

**FIGURE 3:**
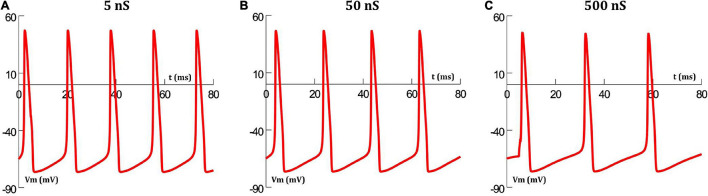
Action potential oscillations observed at the central cell in the 1-D chain of cells, when employing a constant gap junctional mechanism under various levels of gap junctional coupling: **(A)** 5 nS, **(B)** 50 nS, and **(C)** 500 nS. It can be clearly seen that an increase in coupling strength between cells, leads to a reduction in the frequency of oscillations.

**FIGURE 4:**
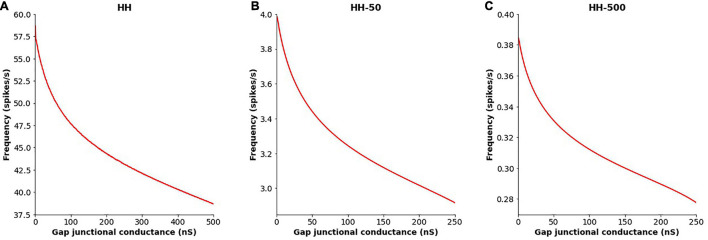
Variation in the frequency of AP oscillations, observed at the central cell in the 1-D chain of cells, with changes in the extent of intercellular coupling with **(A)** HH AP mechanism, **(B)** HH-50 AP mechanism, and **(C)** HH-500 AP mechanism. Similar trends are observed for all these variants, with the oscillation frequency falling with increased gap junctional coupling between cells.

The extent of gap junctional coupling between each pair of adjacent cells in the 1-D model was varied over a wide range of conductance levels. In these simulations, the gap junctions were modeled as passive components where the conductance offered by the gap junctional pathway was constant and independent of the transjunctional voltage between the coupled cells. Figure 3 shows oscillations of HH APs observed at the central cell for 5, 50, and 500 nS coupling strength. The frequencies of oscillations at these levels were 56, 50 and 38 spikes/s, respectively. With increase in the strength of coupling, it is found that the frequency of oscillations falls. The same was explored for HH-50 and HH-500 APs, having wider time courses, and similar trends were observed. Figure 4 shows this variation in frequency for a range of gap junctional coupling strengths.

### Variations in Gap Junction Subtype

Here the constant gap junctional coupling mechanisms employed in the earlier section were replaced with active mechanisms. The conductances offered by various gap junction subtypes have been reported to be sensitive to transjunctional voltage (Desplantez et al., 2007). This involves opening and closing of gates in the protein structure of the gap junctions. Differences have also been reported in the gating kinetics between different subtypes, as well as in their unitary conductances (Desplantez et al., 2007). These characteristics of different subtypes were taken into consideration when developing models for the Cx40, Cx43, and Cx45 gap junction subtypes. To conserve the differences in the unitary conductances (g_*main*_), equal numbers of individual gap junction channels were considered for each subtype (N_*j*_ = 1,000) between each cell pair. The relevant parameters have been presented in Table 2.

**TABLE 2:** Differences in unitary conductances between various gap junction subtypes.

Subtype	g_*main*_ (pS)	N_*j*_	N_*j*_ × g_*main*_ (nS)
Cx40	162	1000	162
Cx43	61	1000	61
Cx45	32	1000	32

*The total number of individual gap junction channels between any two cells was set equal (N_j_ = 1000) for all the subtypes.*

*The values for main state unitary conductances, g_main_, were sourced from Desplantez et al. (2007).*

**FIGURE 5:**
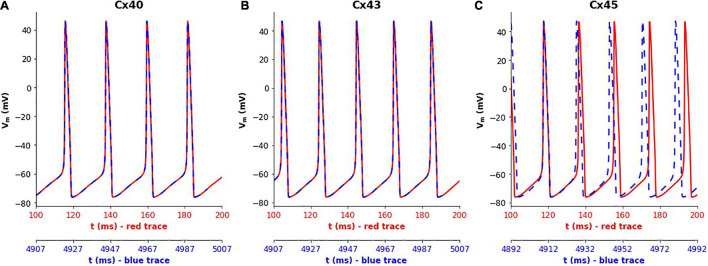
Variations in the spiking frequency observed when simulating the 1-D model for a duration of 5000 ms with different gap junction subtypes. Panels show results for different gap junction subtypes: **(A)** Cx40, **(B)** Cx43, **(C)** Cx45. The traces in red correspond to membrane potential recorded at the central cell during the initial phase of the simulations (around 100–200 ms), while the traces in blue show the same during the final phase of the simulations (around 4,900–5,000 ms). The plots were aligned such as to match the peaks of the first APs in each subplot. It can clearly be seen that the simulations with Cx45 subtype exhibited a rise in the spiking frequency, whereas this remained constant in the case of Cx40 and Cx43.

Simulations of the 1-D model were run for a duration of 5,000 ms for each gap junction subtype. It was found that the frequency of oscillations did not show any noticeable differences in the case of Cx40 and Cx43 subtypes, with the frequency at the central cell remaining constant at 46 and 53 spikes/s, respectively, as seen in Figures 5A,B. But for Cx45, it was seen that the frequency of oscillations increased from 53 (red trace in Figure 5C) to 56 spikes/s (blue trace in Figure 5C) over the course of the simulation. On closer examination, it was found that the oscillation frequency settled to the steady value (of 56 spikes/s) after gradually rising over the first 1,600 ms.

**FIGURE 6:**
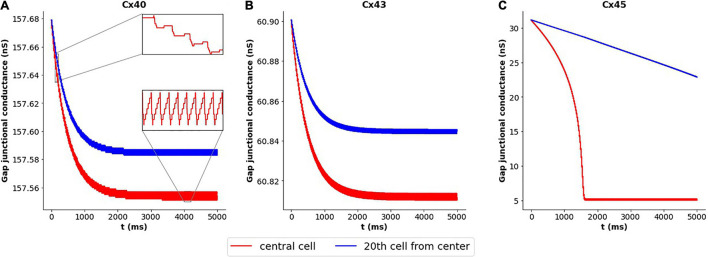
Variation in gap junctional conductance over the course of a simulation, with 1-D model having HH mechanism, while the cell is exhibiting AP oscillations. Panels show results for different gap junction subtypes: **(A)** Cx40, **(B)** Cx43, and **(C)** Cx45. Red trace represents the central cell, while the blue trace represents the 20th cell away from the central cell. The overall change in gap junctional conduction is very low (<0.2%) for Cx40 and Cx43, while Cx45 exhibits a significant fall (∼84%) in gap junctional conductance.

We decided to closer examine the cause of this variation in case of Cx45 subtype, in contrast to Cx40 and Cx43 subtypes. We have seen above how coupling strength influenced the frequency of oscillations. This raised the possibility of changes in gap junctional conductance occurring over the course of our simulations, leading to changes in the exhibited frequency. To explore this, we tracked the gap junctional conductance at the central cell and a distant cell over the course of the entire simulation. Figure 6 plots this coupling strength for Cx40, Cx43, and Cx45. It is seen that the variation in strength of coupling is negligible (<0.2%) for Cx40 and Cx43 subtypes. It should be noted that the unitary conductance (g_*main*_) for Cx40, Cx43, and Cx45 is 162 pS, 61 pS, and 32 pS (Desplantez et al., 2007), respectively, and therefore the maximum conductance of each of the gap junctions is N_*j*_ times their unitary conductances (see Table 2). The inset in Figure 6A shows the detailed variations in gap junctional conductance during repeated spikes. The rise and fall of conductance is an outcome of the continuously varying transjunctional voltage between the connected cells. Initially, the fall is greater than the subsequent rise, but they equalize out over a period of time, and the gap junctional conductance is found to stabilize. However, in the case of Cx45, as seen in Figure 6C, it was found that the gap junctional conductance undergoes a significant fall (∼84%). This occurred during the first 1,600 ms, corresponding to the period of change in oscillation frequency. On closer inspection it was found that the fall in conductance, during repeated APs, was much greater than the subsequent rise. These balanced out only after the gap junctional conductance fell to levels corresponding to the residual state conductance of Cx45 gap junctions. Similar trends were observed for APs with wider time courses, namely, HH-50 and HH-500.

The kinetics of inactivation of Cx40 and Cx43 gap junctional current have been reported to be similar, while Cx45 is slower (Desplantez et al., 2007). It is conceivable that this could contribute to the differences in their emergent behavior, described above, with Cx40 and Cx43 behaving similarly. To test this, the gating kinetics of Cx45 were altered to match that of Cx40 and Cx43. It was found that the oscillations now had a constant frequency of 53 spikes/s, and the gap junctional conductance stabilized after an initial decline. This fall in conductance was very small as compared to that observed previously (1 vs 84%).

### Variations in Size of Syncytium

**FIGURE 7:**
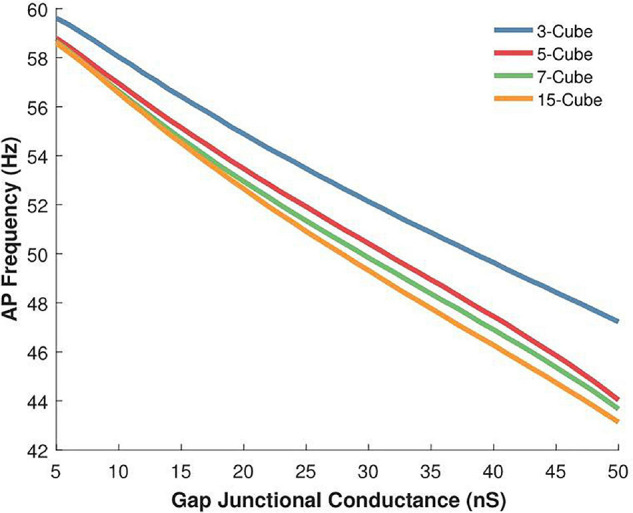
Variations in the action potential oscillation frequency measured in 3-D syncytia of differing sizes for a range of gap junctional conductances (5 to 50 nS). The inverse relation between gap junctional coupling strength and oscillation frequency is observed for the three-dimensional syncytial model, irrespective of its size. For a given coupling strength, the AP firing rate is higher in smaller sized syncytia and this progressively reduces with increase in the size of the syncytium.

In this and the following section, we have re-employed the constant gap junctional coupling mechanism presented earlier. The absence of voltage sensitivity and gating kinetics permits a focused examination of other factors such as the effect of variations in the size of the syncytium, as discussed here. We explored the action potential firing rate for various syncytial sizes, namely 3-, 5-, 7-, and 15- cube, under a range of intercellular coupling strengths. The source code for these simulations have been made available on ModelDB (accession number 244699). In very poorly coupled syncytia, expectedly, gap junctions are incapable of propagating away from the originating cell. Hence, we have excluded the data for weak coupling strengths. The results are summarized in Figure 7. It is seen that the action potential firing rates are higher in smaller sized syncytia, and that the firing rates decay with increasing sizes of the syncytium. As seen earlier, the firing rates are affected by the extent of gap junctional coupling between cells. The variation in AP firing rate between the various syncytial sizes is amplified with enhancement in the intercellular coupling. For example, with the strength of intercellular coupling set at 25 nS, the difference in AP frequency between 3-cube and 15-cube is 4.8%; this rises to 8.7% when the strength is raised to 50 nS.

### Effect of Location of Pacemaking Cell

**FIGURE 8:**
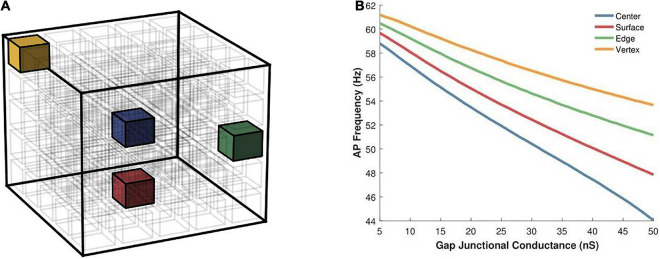
**(A)** An illustration of a 5-cube syncytium showing locations where pacemaker cells were placed (one location per simulation) are highlighted with colored cells, while all other cells have been left uncolored for easier visualization. The bounding box (in black) indicates the extent of the syncytium. **(B)** Action potential oscillation frequency measured in a syncytium of size 5-cube under different locations of the AP initiating cell such as the central cell, a cell on the surface, a cell on an edge, and a cell located at a vertex of the syncytium. Note that the color of the cells in panel **(A)** corresponds to the traces in panel **(B)**.

Having examined the effects of varying the syncytial size in the previous set of simulations, we opted to keep the size of the syncytium constant, at 5-cube, and focus on other factors. Here we begin by exploring the effect of varying the location of the AP initiating cell on the spike oscillation frequency generated by the syncytium. Individual simulations were undertaken with the pacemaker cell located at one of the following: (i) cell at the center of syncytium, (ii) cell at one of the vertices, (iii) cell on one of the edges, and (iv) cell on one of the surfaces. These locations have been illustrated in Figure 8A and the emergent syncytial behavior summarized in Figure 8B. Interestingly, there exists notable differences in the AP firing rate based on the site of initiation of the initial AP. Oscillations initiating at cells with a larger number of coupled neighboring cells, e.g., the central cell, are found to result in a lower syncytial oscillation frequency as compared to oscillations initiating at a relatively sparsely coupled cell, e.g., the vertex cell. These differences are further pronounced when the strength of gap junctional coupling is high. For example, with the strength of intercellular coupling set at 25 nS, the difference in AP frequency between initiation at the center and the vertex is 10.5%; this rises to 21.9% when the strength is raised to 50 nS.

**FIGURE 9:**
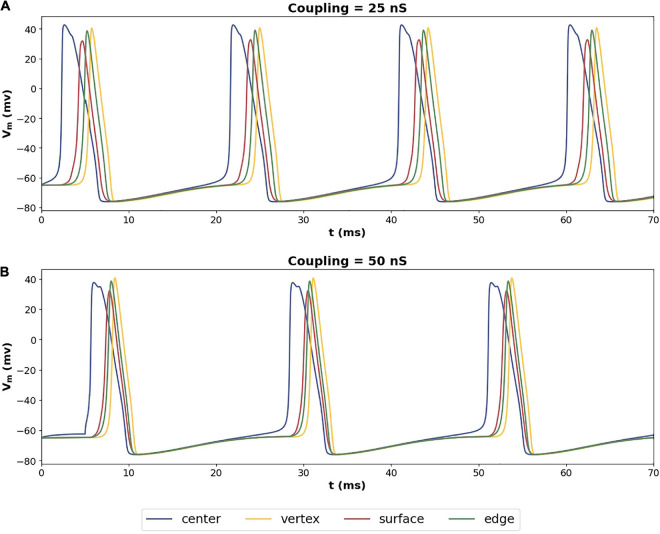
Propagation of action potentials in a syncytium of size 5-cube with an oscillator cell placed at the center of the syncytium. The colors of the traces correspond to the cells highlighted in Figure 8A. Panel **(A)** shows the syncytial response with gap junctional coupling strength of 25 nS, and panel **(B)** shows the same with 50 nS. These values correspond to the mid and max values plotted in Figure 8B.

Exploring the propagation of these APs within the syncytium, its seen that all the cells in the syncytium synchronize to the same frequency of oscillating action potentials, driven by the pacemaking cell. Figure 9 shows the response of a 5-cube syncytium when the pacemaker cell is located at the center, for two different coupling strengths: 25 and 50 nS. Traces for four cells are shown in each plot: the central cell, a cell located at the vertex, a cell located on a surface, and a cell located on an edge. It can be seen that all the cells oscillate with the same frequency. For gap junctional coupling of 25 nS, this frequency was 51.9 spikes/s, while at an increased gap junctional coupling of 50 nS, this frequency dropped to 44.0 spikes/s. The source code for these simulations have been made available on ModelDB (accession number 244699).

### Effect of Multiple Pacemaking Cells

**FIGURE 10:**
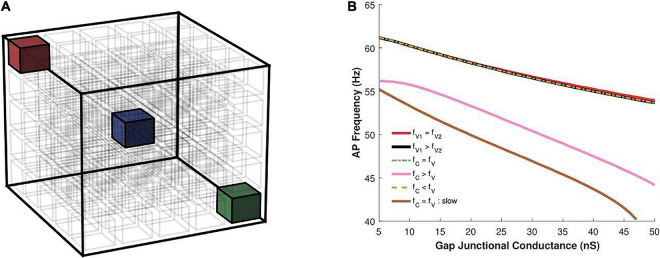
**(A)** An illustration of a 5-cube syncytium showing locations where pacemaker cells were placed (two locations per simulation) are highlighted with colored cells, while all other cells have been left uncolored for easier visualization. The bounding box (in black) indicates the extent of the syncytium. **(B)** Action potential oscillation frequency measured in a syncytium of size 5-cube under presence of two oscillator cells in the syncytium at varying locations, as shown in panel **(A)**, with the two cells individually producing oscillations at either the same or different frequencies. f_*V*_, frequency of oscillator at vertex; f_*C*_, frequency of oscillator at center. The legend indicates how their oscillation frequencies compare for each trace. Note that the color of the cells in panel **(A)** do not correspond to the traces in panel **(B)**, and is only used to for clarity of description in the text.

Multiple propagating spikes can be generated simultaneously in a syncytium, resulting from the distributed ground-plexus nature of the innervation and the possibility of co-activation of neurotransmitter release at two or more synapses. In order to explore the effects of this feature of autonomic neurotransmission, we investigated the effect of having multiple oscillators within the same syncytium. The size of the syncytium, as in the previous study, was kept as 5-cube. We tested two possibilities: (i) both oscillators possessing the same frequency, and (ii) oscillators possessing different frequency. The oscillators were placed either centrally or at the vertices, as illustrated in Figure 10A, as these locations were found above to exhibit maximal variations. When both oscillators were placed at the vertices (red and green cell in Figure 10A), these were positioned at the opposite extremes. The slower oscillator introduced here was implemented by setting the peak sodium conductance to a smaller value (0.4 S/cm^2^) than that for the faster oscillator (0.75 S/cm^2^); the latter having been employed in previous simulations of the 3D syncytial model. The resultant uncoupled oscillator frequencies were 58 and 63 spikes/s, respectively. Figure 10B presents the results obtained under the various configurations. When the two oscillators fired at equal frequencies (both fast) and were located at the vertices (f_*V1*_ = f_*V2*_), the resultant oscillation frequency was similar to that when a single such oscillator was located at a vertex. Next, we moved one of the oscillators to the center of the cubic lattice layout (blue cell in Figure 10A). In this scenario (f_*C*_ = f_*V*_), though both the oscillators are identical, their location within the syncytium endows them with different properties. The central oscillator will result in slower syncytial oscillations than the vertex oscillator, and the syncytium synchronizes with the latter. A similar behavior was observed when two vertex oscillators were endowed with differing oscillation frequencies (f_*V1*_ > f_*V2*_); here the syncytium synchronized in step to the faster oscillator. The same is observed when the central oscillator is slower than the vertex oscillator, wherein the syncytium synchronizes with the faster oscillator located at the vertex. In this case, the outcome is compounded by the central location of the oscillator; as seen in Figure 8B, oscillators located centrally exhibit lowered AP frequency than when placed at the vertices. The configuration that produces markedly different syncytial behavior is when the central oscillator is faster than the vertex oscillator (f_*C*_ > f_*V*_). Here the syncytium attains a much lower oscillation frequency corresponding to that for a single central oscillator. If both the oscillators were slow (f_*C*_ = f_*V*_ : slow), the resulting oscillatory behavior would have been even lower. This confirms that the higher spiking rate of the central oscillator in f_*C*_ > f_*V*_ allows it to dominate the syncytial behavior.

## Discussion

Gap junctions are known to influence the electrical responses of cells. Electrical activity of cells in an electrical syncytium is not just determined by the properties of the cells individually, but by a number of other syncytial parameters (Appukuttan et al., 2015a). In this study we have attempted to explore the effect of variations in gap junctional coupling on the frequency of electrical oscillations in syncytial tissues. The results presented here clearly show that the nature of gap junctional coupling affects the syncytial response. The spike oscillation frequency decreases with an increase in the strength of coupling. This may be explained in terms of charge retention and dissipation during an AP. Under increased intercellular coupling, the gap junctional shunts are more effective, thereby dissipating a larger proportion of the charge to neighboring cells. This results in a slower build-up of charge within a cell, thus requiring a more prolonged depolarization phase to attain threshold for producing an AP. On the other hand, under low levels of gap junctional coupling there is greater charge retention within cells, leading to faster depolarization to threshold. These trends were further verified using action potentials of widely varying time courses, eliminating AP profile as a possible determinant of the observed differences.

Our results also suggest a difference in oscillatory behavior based on the gap junction subtype involved in intercellular coupling. Some of the differences could be attributed to the differences in the unitary conductances. On further investigation, it was observed that gap junctional gating kinetics also played a role in determining the frequency of oscillation. Most modeling studies tend to employ constant gap junctional coupling. As demonstrated here, this might not always be an appropriate choice. Our findings strongly support the proposition that gap junction subtypes are physiologically unique, and often one subtype cannot be adequately replaced by another subtype. This is exemplified in certain pathologies, such as that of the failing human ventricular tissue, where the downregulation of Cx43 is accompanied by elevated levels of Cx40 and Cx45 (Desplantez et al., 2007).

For very low levels of gap junctional coupling, an AP elicited at a cell within the syncytium is unable to propagate to its neighboring cells. With increase in the intercellular coupling, these APs begin to propagate to other cells, and spread through the syncytium. If any cell in a well coupled syncytium acts as a pacemaker, i.e., produces oscillations of APs, then these permeate through the entire syncytium leading to synchronized oscillations across all cells. The resultant oscillation frequency certainly depends on the inherent frequency of the pacemaking cell, but is also governed by factors such as the size of the syncytium and the relative location of the pacemaking cell within the syncytium. The observation that the pacemaking activity is weaker when the cell is located centrally rather than at the vertices, aligns well with past studies which have shown the latter to endow higher excitability to the cells (Appukuttan et al., 2017).

### Limitations and Extensions to Study

The use of a “standard” reference AP mechanism was deemed necessary to help delineate the syncytial contributions, as far as possible, of ion channels, gap junctions and the syncytial interplay. The classical HH model, being a well understood AP model, was therefore employed in this study to explore this computationally. This also allowed for better assimilation and integration of knowledge gathered from our prior syncytial studies wherein the HH models were effectively employed. Other alternatives for a “standard” reference AP mechanism include modifications of the HH model such as the Morris-Lecar model (Morris and Lecar, 1981), or more recent models such as that by Herrera-Valdez and Lega (2011).

The detrusor smooth muscle has been found to exhibit diverse AP profiles, and DSM cells are known to possess an array of nine or more active channels contributing to their generation (Brading, 2006; Steers and Tuttle, 2009; Brading and Brain, 2011; Petkov, 2011). Some of us have previously published a study where we report the development of the full complement of ion channels necessary to accurately reproduce the single cell action potentials (Mahapatra et al., 2018). Currently, we are working toward combining this with a passive DSM syncytial model (Appukuttan et al., 2015a) to achieve biological realism and arrive at experimentally testable predictions. But pilot studies have shown that in view of the complexity of these models—the involvement of various ions, a large ensemble of channels, diverse gating mechanisms (e.g., voltage-gated, Ca^2+^-activated, ligand-gated), calcium dynamics, gap junction subtypes with their own specific gating properties and kinetics—it is infeasible to identify the contributions of these disparate mechanisms without peeling away some of this complexity. However, certain advances in understanding can be achieved by setting certain parameters to constants, e.g., the gap junctional conductance (i.e., constant gap junctional coupling), or using simplified sub-models, e.g., the HH AP mechanism. Neither of these represent biological reality accurately, but serve to provide insights into the building blocks of complex behavior. The present study is one such example.

The current study employed only homomeric-homotypic gap junction subtypes. One reason for this was the availability of pertinent experimental info in literature for the development of models for these subtypes. Quantitative studies pertaining to heterotypic gap junctions in the detrusor or cardiac tissue are comparatively sparse (Desplantez et al., 2007). Another reason for not exploring homomeric-heterotypic or heteromeric-homotypic or heteromeric-heterotypic gap junctions, was the lack of availability of a computational model for these that could be employed in our study. We have in the past attempted to model homomeric-heterotypic gap junctions, but met with limited success (Appukuttan et al., 2015b). Future studies could attempt to develop appropriate models for these based on data from recent experimental studies (Willy et al., 2017; Santos-Miranda et al., 2019). Further, it will be essential to have gap junction models that are capable of allowing bi-directional flow of Ca^2+^ ions, and such models are currently unavailable. Theoretical conceptualization of these models is straightforward, but their implementation for simulators has been difficult, and is an area of ongoing work (Sengupta and Manchanda, 2020).

It should also be noted that the gap junctional models employed here need to be further improved upon and awaits the availability of more detailed experimental data in regards to their physiology. Data underlying the gap junction models employed here were obtained *via* digitization of available published figures, and this commonly involves issues related to poor resolution of data. For a more definitive understanding of different gap junction subtypes, it is essential to obtain higher resolution data about their voltage sensitivity and kinetics, across a wide range of transjunctional voltages, thereby also eliminating any need to extrapolate data.

In the presence of multiple pacemaking cells, it was seen here that the syncytium gets entrained to the highest frequency, as has been reported previously (Parsons and Huizinga, 2016). It would be useful to undertake a detailed study of interplay between coupled oscillators in a smooth muscle syncytium, with the interstitial cells of Cajal (ICC), interspersed between smooth muscle cells (SMCs), acting as the pacemaking centers. Past studies have explored interactions between ICCs based on the coupled oscillator theory to evaluate emergent network properties (Parsons and Huizinga, 2015; Wei et al., 2017). These could be investigated in the context of an inhomogeneous syncytium with some SMCs in the current model being replaced by ICCs. Experimental studies have reported the presence of pacemaking activity in syncytial tissues such as the detrusor (Meng et al., 2008). Their origin in DSM is still unclear, although certain studies have proposed that they are engendered by interstitial cells of Cajal, as identified for the gastrointestinal tract (Sanders et al., 2006), while other studies have suggested a myogenic origin (Meng et al., 2008). Electrophysiological recordings have shown pacemaker-type APs in the DSM, with their preponderance in recordings obtained from hyperactive cells.

The study presented here serves to indicate various factors that influence the emergent oscillatory response in syncytial networks, such as the characteristics of the gap junctions involved, in terms of their conductances, sensitivity to transjunctional voltage and gating kinetics, the size of the syncytium, and the location of the oscillator cells within this syncytium. The presented results advocate that it is pertinent to identify the complement of gap junction subtypes forming the syncytium, and to conduct targeted investigations of their biophysical properties and related syncytial factors. While biological realism is a desirable feature of models, the attainment of such realism in the model for any particular cell or tissue is an incremental process, different degrees of realism being found at different stages of development of the model. Moreover, the degree of realism is closely related to the questions being addressed. We look forward to working with more biologically realistic models in the future, once the foundations have been laid with the exploratory work, such as that reported here. We are hopeful that the results presented here will propound more focused research on various aspects of syncytial interactions, such as the biophysical differences between the underlying gap junction subtypes, and the nature of pacemaking activity in the syncytium, and contribute toward a better understanding of their physiology.

## Data Availability Statement

The raw data supporting the conclusion of this article will be made available by the authors, without undue reservation.

## Author Contributions

SA designed the computational studies, performed the simulations, analyzed the data and wrote the first draft of the manuscript. KB and RM contributed toward the interpretation of the results, designing of simulations and revision of the manuscript. All authors contributed to the article and approved the submitted version.

## Conflict of Interest

The authors declare that the research was conducted in the absence of any commercial or financial relationships that could be construed as a potential conflict of interest.

## Publisher’s Note

All claims expressed in this article are solely those of the authors and do not necessarily represent those of their affiliated organizations, or those of the publisher, the editors and the reviewers. Any product that may be evaluated in this article, or claim that may be made by its manufacturer, is not guaranteed or endorsed by the publisher.
