# Supplementary material for: Effect of Variations in Gap Junctional Coupling on the Frequency of Oscillatory Action Potentials in a Smooth Muscle Syncytium
Source: Front Physiol. 2021 Oct 1;12:655225. doi: 10.3389/fphys.2021.655225 (PMC8517141; doi:10.3389/fphys.2021.655225)
Supplement: Supplementary file 1 [file Data_Sheet_1.pdf]

Effect of Variations in Gap Junctional Coupling on the Frequency of Oscillatory Action Potentials in a Smooth Muscle Syncytium

Shailesh Appukuttan[1*], Keith L. Brain[2], Rohit Manchanda[1]

[1] Department of Biosciences and Bioengineering, Indian Institute of Technology Bombay, Mumbai, India

[2] Institute of Clinical Sciences, College of Medical and Dental Sciences, University of Birmingham, United Kingdom

*** Correspondence:** Shailesh Appukuttan (shailesh.a@iitb.ac.in)

Supplementary Document

S1. Compartmental Modeling & NEURON Simulator

Neurons are often modeled in terms of their electrical equivalent circuits. The basis of this modeling lies in the representation of the cell membrane as a parallel combination of a resistor and capacitor, often termed as a RC circuit. The resistor corresponds to the conductance of the leak channels in the cell membrane. A battery is placed in series with this resistor, and corresponds to the reversal potential of these leak channels. To study the variations in membrane potential across a spatially distributed region, as opposed to just a point neuron model, we connect multiple such as RC circuits together, with parameters for the electrical elements adjusted as required (Sterratt, et al., 2011). For a more detailed discussion of the compartmental modeling technique, readers are suggested to refer to chapter 4 of Sterratt, et al. (2011). Figure 4.1 in Sterratt, et al. (2011) provides a useful illustration on how a neuronal structure is modeled via the compartmental modeling technique.

Other ion channels, such as the Hodgkin-Huxley Na^+ and K^+ channels, can be accommodated by adding more segments in parallel with the RC arrangement described above. Fig. 1d (of the manuscript) shows how the smooth muscle cells in our model are modeled using this approach. Each cell in our model comprises of 51 compartments (details in section S2), with each compartment being translated into its electrical equivalent circuit. The equations underlying the transmembrane current (I_m) in each individual compartment are shown below:

$$I_m = I_C + I_{ionic} = I_C + I_{Na} + I_K + I_{leak} \quad \text{(eq. S1)}$$

$$I_C = C_m \frac{dV_m}{dt} \quad \text{(eq. S2)}$$

$$I_{Na} = g_{Na} \times \left(V_m - E_{Na}\right) \quad \text{(eq. S3)}$$

$$I_K = g_K \times (V_m - E_K) \quad \text{(eq. S4)}$$

$$I_{leak} = g_{leak} \times (V_m - E_{leak}) \quad \text{(eq. S5)}$$

where V_m is the membrane potential of that compartment; C_m is the capacitance offered by the lipid bilayer forming the cell membrane and Ic is the resultant capacitive current; I_{Na}, I_K, I_{leak} are the currents attributed to the sodium, potassium and leak channels, respectively, and E_{Na}, E_K and E_{leak} correspond to the reversal potentials of these channels.

In addition to this, the law of conservation of charge dictates that the summation of all currents flowing into any region of a neuron must equal zero (Hines & Carnevale, 1997). With respect to the bottom panel of fig. 1d (of the manuscript), applying the law of conservation of charge to the middle compartment gives us:

$$\sum i_a - \int_A i_m dA = 0 \quad \text{(eq. S6)}$$

where Σi_a represents the summation of all axial currents i_a (mA) flowing into that compartment (from its neighboring compartments), and the second term represents the total transmembrane current, evaluated by integrating the transmembrane current density i_m (mA/cm^2) over the total membrane area of that compartment A (cm^2). For a more detailed discussion, readers are suggested to refer to chapter 3 of Hines & Carnevale (1997).

The NEURON simulation environment is one of the most widely used simulators in the field of computational neuroscience. It employs the above described compartmental modeling technique, alongside other foundational electrophysiological formalisms such as the Goldman equations, the Hodgkin-Huxley model, and cable theory. It enables modelers to describe and develop their models in terms of structural units and biophysical parameters. It then internally generates the equations corresponding to the electrical equivalent circuit of the resultant model, and evaluates these equations as required during the running of simulations.

S2. Modeling Detrusor Smooth Muscle Syncytia

Modeling in our study was undertaken on the NEURON simulator (Hines & Carnevale, 1997) using the compartmental modeling technique (Bower & Beeman, 2012). Individual detrusor smooth muscle (DSM) cells were modeled as thin, long cylinders with a length of 200 µm and diameter of 6 µm. Each cell was modeled so as to consist of 51 compartments, which translates to each compartment being 3.92 um wide. Our previous studies have demonstrated that this offers ample resolution for evaluating cable properties. The larger the number of segments per cell, the greater is the spatial resolution achieved. But this is at the expense of increased computational load, which translates into much longer run times. Our choice of 51 segments for the DSM cells modeled here, is much greater than that determined by NEURON's built-in "d_lambda" criterion (nseg = 5) (Appukuttan et al., 2015a).

This is a provisional file, not the final typeset article

Some of the other biophysical parameters employed in the model are listed in Table S1. Parameter values were selected on the basis following a survey of relevant literature - see Appukuttan et al., (2015a) for more details.

Parameter	Value
Cell Length	200 um
Cell Diameter	6 um
Resting Membrane Potential	-50 mV
Axial Resistivity	183 Ω.cm
Membrane Resistivity	132.5 kΩ.cm^2
Specific Membrane Capacitance	1 uF/cm^2

Table S1: Values of parameters employed in model development.
Source: Appukuttan et al. (2015a)

Gap junctions were modeled as resistive pathways, as illustrated in Fig. 1b of the manuscript. In the NEURON simulator, the bi-directional gap junctional coupling between two cells is implemented by means of a pair of unidirectional point process mechanisms, one hosted by each cell. In the original model, the conductance of the gap junctions was kept constant during a simulation. In the current study, certain simulations involved following the variation of the gap junctional conductance during the course of the simulation. The development of these gap junction models is described in the following section.

Using the developed gap junctional mechanism, cells were coupled together to form networks with different layouts. The current study employed two layouts:

- **1-D model:** 181 cells were coupled to form a chain of DSM cells. This resulted in every cell having two neighboring cells to which it was coupled intracellularly by means of gap junctions; except for the two cells at the terminal ends, which only had a single neighboring cell. This is illustrated in Fig. 1a.

- **3-D model:** A three-dimensional layout was achieved in the form of a cubic lattice arrangement of cells. Each DSM, in the interior of the syncytium, is coupled to six other cells; two along each axes. As they are cubes, their sizes were denoted in terms of the number of cells along any edge. E.g. 5-cube refers to a syncytium of 125 cells arranged 5 x 5 x 5. In this case, there are 27 cells in the interior that have six neighboring cells, while the other cells have fewer neighbors as determined by their locations – the 8 cells at the vertices having the least, with just 3 neighboring cells each.

S3. Modeling of Gap Junctions

Gap junctions are implemented in NEURON by means of point process mechanisms to implement localized conductances. This basically means that their effect is localized to the compartments that they are attached. As illustrated in fig. 1b of the manuscript, a bidirectional gap junction between two cells is implemented in NEURON by means of a set of two such point processes per paired cells. A point process is attached to each of the two paired cells, whilst also keeping track of the membrane potential of the other cell. Thereby, each point process is able to independently evaluate the transjunctional voltage, and thereby determine the gap junctional current that should flow in/out of its host cell. The value of gap junctional current is determined at every time step by the following equation:

$$I_j^n = (V_m^n - V_m^{n+1}) \times G_j \qquad \text{(eq. S7)}$$

where I_j^n denotes the gap junctional flowing in/out of cell n, V_m^n and V_m^{n+1} are the membrane potentials of cell n and cell $n+1$, respectively, and G_j is the gap junctional conductance between the two cells.

Experimental studies in the past have reported individual gap junction channels to exist in one of the following states (Bukauskas et al., 1995):

- **Main state:** a voltage-dependent maximum conductance state (g_{main})
- **Residual state**: a voltage-independent low conductance state ($g_{residual}$)
- **Closed state**: a non-conducting state

Gap Junction Subtype	g_{main} (pS)	$g_{residual}$ (pS)
Cx40	162	28
Cx43	61	12
Cx45	32	12

Table S2: Unitary conductances of various gap junction subtypes employed in study.
Source: Desplantez et al. (2007)

Apart from these, the gap junction channels can also exist in multiple short-lived sub-conductance states between the open and residual state (see Figs. 2, 3 in Bukauskas et al., 1995). Table S2 lists the values of main state and residual state conductance levels for the various gap junction subtypes explored in this study.

Past studies have also reported the dependence of the extent of gap junctional coupling between two cells on the transjunctional voltage between the two coupled cells (Desplantez et al., 2004, Desplantez et al., 2007). Fig. 5 in Desplantez et al. (2007) shows plots of normalized gap junctional conductance levels as a function of the transjunctional voltage (V_j). This data was digitized, multiplied by the

This is a provisional file, not the final typeset article

maximum unitary conductances as indicated by the main state conductance level (g_{main}), and then curve-fitted to obtain mathematical functions that provided best fit to this data.

In brief, as outcome of the fitting procedure, the dependence of gap junctional conductance to transjunctional voltage for each hemi-channel could be represented by a Boltzmann equation (see Appukuttan et al., 2015b for more details), reflecting its non-ohmic response:

$$g_{left} = \frac{g_{max_{left}} - g_{min_{left}}}{1 + exp\left(\frac{-v_j - V_{0_{left}}}{S_{left}}\right)} + g_{min_{left}} \qquad \text{(eq. S8)}$$

$$g_{right} = \frac{g_{max_{right}} - g_{min_{right}}}{1 + exp\left(\frac{v_j - V_{0_{right}}}{S_{right}}\right)} + g_{min_{right}} \qquad \text{(eq. S9)}$$

where g_{left} and g_{right} are the conductances offered by the two hemi-channels individually. The parameters used for fitting these equations to the various subtypes are listed in Table S3.

**Gap Junction Subtype**	**g_{max}**	**g_{min}**	**S**	**V_0**
Cx40	324	28.67	13.83	48.43
Cx43	122	17.30	8.94	55.99
Cx45	64	5.37	9.36	32.86

Table S3: Parameters used for fitting eqs. S7 & S8 for various gap junction subtypes.
Source: Appukuttan et al. (2015b)

Using this approach, individual mathematical models were developed for representing the dependence of gap junctional conductance on transjunctional voltage for hemi-channels of Cx40, Cx43 and Cx45 gap junction subtypes. As the current study solely employs homomeric-homotypic gap junctions, where both the hemi-channels can be assumed to be identical, we have used the same parameters (from table S3) for left and right hemi-channels of each gap junction subtype.

As the two hemi-channels are arranged in an anti-serial arrangement within a gap junction, the net gap junctional conductance, g_j, between two coupled cells can now be evaluated as:

$$g_j = \frac{g_{left} \times g_{right}}{g_{left} + g_{right}} \quad \text{(eq. S10)}$$

The total gap junctional current, I_j, flowing between two coupled cells can then be evaluated by:

$$I_j = V_j \times g_j \times N_j = V_j \times G_j \quad \text{(eq. S11)}$$

where V_j is the transjunctional voltage, g_j is the voltage-dependent single channel conductance, N_j is the total number of individual gap junction channels between two coupled cells, and G_j is the total gap junctional conductance between them.

Gap junctional coupling is known to be not just voltage-dependent, but also a function of time. This is evident in the difference between instantaneous conductance levels and steady-state conductance levels following a change in the transjunctional voltage. See fig. 5 in Desplantez et al. (2007) for experimental recordings highlighting this difference.

The kinetics of this change in conductance level on change in transjunctional voltage is commonly characterized using the time constant of change from the steady state conductance level at one transjunctional voltage to the steady state conductance level at another transjunctional voltage; the steady state conductance levels having being determined as described above. This time constant has been experimentally evaluated and reported for Cx43 and Cx45 in past studies (Fig. 5B in Lin et al., 2003; Fig. 6A in Moreno et al. 1995). Desplantez et al. (2007) reported that the kinetics of Cx40 and Cx43 were similar, and faster than that of Cx45. Similar to the curve fitting process described earlier, this time constant of decay was modeled by fitting to a Gaussian distribution:

$$\tau = a \times exp\left[-\left(\frac{V_j - b}{c}\right)^2\right] \quad \text{(eq. S12)}$$

where V_j is the transjunctional voltage, a is the height of the curve's peak, b is the position of the center of the peak and c represents the standard deviation that determines the width of the bell-shaped Gaussian curve.

Fig. S1 shows graphically both the digitized data and the curves fitted to these. The fits in both cases had a R-squared value of > 0.98. The parameters for eq. S12 derived from the fitting process, for each gap junction subtype, is summarized in Table S4.

This is a provisional file, not the final typeset article

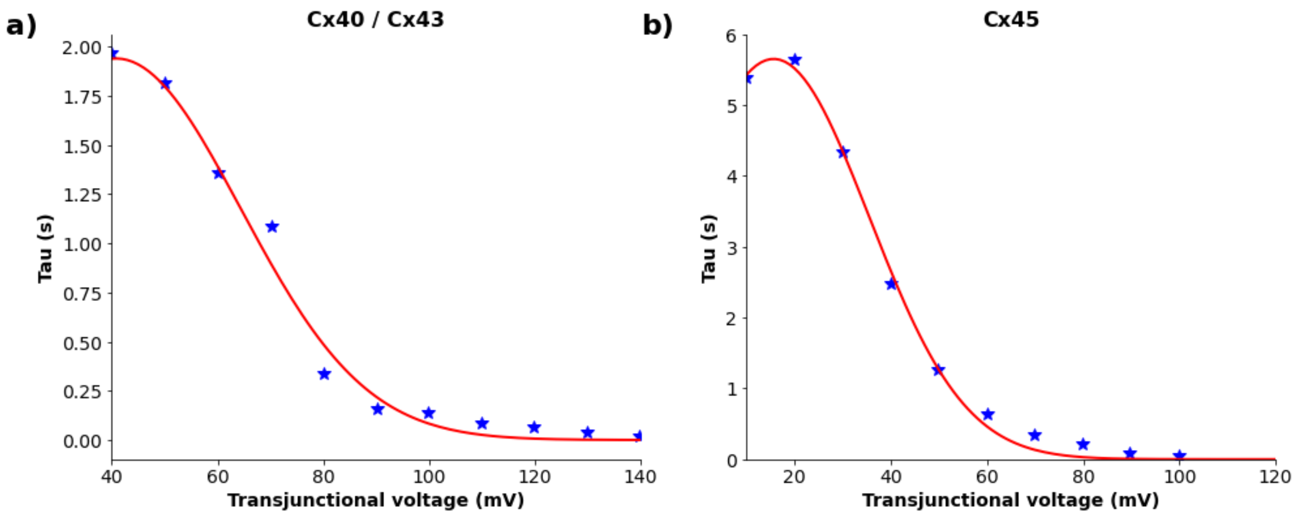

Figure S1: Curves fitted to experimental data using a Gaussian equation for the different gap junction subtype time constants. The blue markers show the data digitized and extracted from Lin et al. (2003) and Moreno et al. (1995), while the red curves indicate eq. S12 with parameters listed in Table S4.

Gap Junction Subtype	a	b	c	R^2
Cx40	1.94	40.83	33.21	0.99
Cx43	1.94	40.83	33.21	0.99
Cx45	5.65	15.63	28.05	0.99

Table S4: Parameters providing best fit to eq. S12 for gap junction subtypes.
Adapted from: Appukuttan et al. (2016)

It should be noted that the gap junction model, developed as described above and employed in our study, does not correspond to individual gap junctional channels between cells, but rather the population of gap junctional channels between two electrically coupled cells. This can be seen in equation S11 where the total number of individual gap junctional channels is taken into account via parameter N_j. Hence, we are modeling a population behavior and focusing on the total gap junctional current flowing between two cells. The model itself therefore does not directly incorporate the various gap junction conductance states, but the emergent total conductance takes this into account via the mathematical fitting procedure discussed above. The gap junctional models developed through the above discussed approach are dependent both on transjunctional voltage and time.

References

Appukuttan, S., Brain, K., & Manchanda, R. (2015a). A computational model of urinary bladder smooth muscle syncytium. Journal of Computational Neuroscience, 38, 167-187.

Appukuttan, S., Sathe, R., & Manchanda, R. (2015b). Modular approach to modeling homotypic and heterotypic gap junctions. In 2015 IEEE 5th International Conference on Computational Advances in Bio and Medical Sciences (ICCABS) (pp. 1-6). IEEE.

Bower, J. M., & Beeman, D. (2012). The book of GENESIS: exploring realistic neural models with the GEneral NEural SImulation System. Springer Science & Business Media.

Bukauskas, F. F., Elfgang, C., Willecke, K., & Weingart, R. (1995). Biophysical properties of gap junction channels formed by mouse connexin40 in induced pairs of transfected human HeLa cells. Biophysical journal, 68(6), 2289-2298.

Desplantez, T., Halliday, D., Dupont, E., & Weingart, R. (2004). Cardiac connexins Cx43 and Cx45: formation of diverse gap junction channels with diverse electrical properties. Pfl{\"u}gers Archiv, 448, 363-375.

Desplantez, T., Dupont, E., Severs, N. J., & Weingart, R. (2007). Gap junction channels and cardiac impulse propagation. Journal of Membrane Biology, 218(1), 13-28.

Hines, M. L., & Carnevale, N. T. (1997). The NEURON simulation environment. Neural computation, 9(6), 1179-1209.

Lin, X., Crye, M., & Veenstra, R. D. (2003). Regulation of connexin43 gap junctional conductance by ventricular action potentials. Circulation research, 93(6), e63-e73.

Moreno, A. P., Laing, J. G., Beyer, E. C., & Spray, D. C. (1995). Properties of gap junction channels formed of connexin 45 endogenously expressed in human hepatoma (SKHep1) cells. American Journal of Physiology-Cell Physiology, 268(2), C356-C365.

Plonsey, R., & Barr, R. C. (2007). Bioelectricity: a quantitative approach. Springer Science & Business Media.

Sterratt, D., Graham, B., Gillies, A., & Willshaw, D. (2011). Principles of computational modelling in neuroscience. Cambridge University Press.

This is a provisional file, not the final typeset article
